# Successful Implementation of a Multicountry Clinical Surveillance and Data Collection System for Ebola Virus Disease in West Africa: Findings and Lessons Learned

**DOI:** 10.9745/GHSP-D-16-00186

**Published:** 2016-09-28

**Authors:** Reshma Roshania, Michaela Mallow, Nelson Dunbar, David Mansary, Pranav Shetty, Taralyn Lyon, Kacey Pham, Matthew Abad, Erin Shedd, Anh-Minh A Tran, Sarah Cundy, Adam C Levine

**Affiliations:** a International Medical Corps, Los Angeles, CA, USA; b Ministry of Health, Monrovia, Liberia; c International Medical Corps Sierra Leone, Freetown, Sierra Leone; d The Warren Alpert Medical School of Brown University, Providence, RI, USA

## Abstract

**Background:**

The 2014 outbreak of Ebola virus disease (EVD) in West Africa was the largest ever recorded. Starting in September 2014, International Medical Corps (IMC) managed 5 Ebola treatment units (ETUs) in Liberia and Sierra Leone, which cumulatively cared for about 2,500 patients. We conducted a retrospective cohort study of patient data collected at the 5 ETUs over 1 year of operations.

**Methods:**

To collect clinical and epidemiological data from the patient care areas, each chart was either manually copied across the fence between the high-risk zone and low-risk zone, imaged across the fence, or imaged in the high-risk zone. Each ETU’s data were entered into a separate electronic database, and these were later combined into a single relational database. Lot quality assurance sampling was used to ensure data quality, with reentry of data with high error rates from imaged records.

**Results:**

The IMC database contains records on 2,768 patient presentations, including 2,351 patient admissions with full follow-up data. Of the patients admitted, 470 (20.0%) tested positive for EVD, with an overall case fatality ratio (CFR) of 57.0% for EVD-positive patients and 8.1% for EVD-negative patients. Although more men were admitted than women (53.4% vs. 46.6%), a larger proportion of women were diagnosed EVD positive (25.6% vs. 15.2%). Diarrhea, red eyes, contact with an ill person, and funeral attendance were significantly more common in patients with EVD than in those with other diagnoses. Among EVD-positive patients, age was a significant predictor of mortality: the highest CFRs were among children under 5 (89.1%) and adults over 55 (71.4%).

**Discussion:**

While several prior reports have documented the experiences of individual ETUs, this study is the first to present data from multiple ETUs across 2 countries run by the same organization with similar clinical protocols. Our experience demonstrates that even in austere settings under difficult conditions, it is possible for humanitarian organizations to collect high-quality clinical and epidemiologic data during a major infectious disease outbreak.

## Abstract (precis)

Despite resource and logistical constraints, International Medical Corps cared for thousands at 5 Ebola treatment units in Liberia and Sierra Leone between 2014 and 2015 while collecting hundreds of data points on each patient. To facilitate data collection and global reporting in future humanitarian responses, standardized data forms and databases, with clear definitions of clinical and epidemiological variables, should be developed and adopted by the international community.

## INTRODUCTION

The outbreak of Ebola virus disease (EVD) in West Africa that began in 2014 is the largest since the Ebola virus was first discovered in 1976. Nearly 30,000 people were infected and almost 12,000 died in the hardest-hit countries: Liberia, Sierra Leone, and Guinea.^1^^-^^3^ The World Health Organization (WHO) formally declared the Ebola epidemic in West Africa a public health emergency of international concern on August 8, 2014.^4^ Days later, International Medical Corps (IMC), which had already begun its own assessment, launched its initial response to the outbreak.

Starting in September 2014, IMC opened and managed 5 Ebola treatment units (ETUs) in Liberia and Sierra Leone. These 5 ETUs cumulatively cared for more than 2,500 patients. IMC used a comprehensive approach to EVD prevention and management, which included direct health care within ETUs; water, sanitation, and hygiene interventions; psychosocial support; support for infection, prevention, and screening in local health facilities; and social and behavior change elements within affected and at-risk communities.

From the 5 ETUs in Liberia and Sierra Leone, IMC amassed more than 25,000 pages of clinical, epidemiological, psychosocial, and operational data over the course of the epidemic. IMC established an Ebola Research Team in March 2015 with the goal of collecting, aggregating, cleaning, quality checking, and analyzing this data to better inform the scientific and humanitarian response to future epidemics.

IMC opened and managed 5 Ebola treatment units in Liberia and Sierra Leone, which together cared for more than 2,500 patients.

The goal of IMC’s Ebola Research Team was to collect and analyze quality data to better inform responses to future epidemics.

Several prior reports have documented the experiences of individual ETUs in Sierra Leone and Guinea. However, no prior studies have presented data from multiple ETUs across multiple countries run by the same organization with similar clinical protocols.^5^^-^^9^ In addition, prior published studies have focused on demographic and outcome data for patients with EVD, and have not presented a comprehensive picture of the details involved in both providing and documenting clinical care for patients with EVD in resource-limited settings.

This study presents IMC’s EVD case management operations across Liberia and Sierra Leone, including numbers and trends of patient admissions to our ETUs; key demographic information and outcomes among admitted patients; and geographical and longitudinal displays of patient admissions, EVD positivity, and mortality. In addition, we provide detailed information, within this article and the supplemental appendices, on the clinical care provided to patients and the methods of data collection within our ETUs.

This study provides detailed information on ways to both provide and document clinical care in a resource-limited setting.

## METHODS

### Study Design

This retrospective cohort study includes patient data collected at 5 ETUs operated by IMC in Liberia and Sierra Leone between September 15, 2014, and September 15, 2015, as part of IMC’s comprehensive response to the West African EVD epidemic. Ethical approval for this study and exemption from informed consent was provided by the Sierra Leone Ethics and Scientific Review Committee, the University of Liberia–Pacific Institute for Research and Evaluation Institutional Review Board, and the Lifespan Rhode Island Hospital Institutional Review Board.

### Program Setting

In cooperation with local health ministries, IMC operated 5 ETUs in Sierra Leone and Liberia between September 15, 2014, and December 31, 2015. The first 2 ETUs to open, in September and November 2014, were located in Bong County and Margibi County, respectively, in Liberia, where the epidemic first peaked in the summer and fall of 2014. As the epidemic began to peak in neighboring Sierra Leone, 2 additional ETUs were established there: in Lunsar, Port Loko District, and in Makeni, Bombali District. In April 2015, IMC assumed management of a fifth ETU in Kambia, Kambia District, Sierra Leone.

### Patient Triage Procedures

Individuals experiencing symptoms consistent with EVD arrived at IMC’s 5 ETUs in 3 ways: transported in an IMC ambulance, transported in a government or private ambulance, or via their own means of transportation (private car, taxi, or walking). The Liberia ETUs received all patients from the ETUs’ catchment areas. In Sierra Leone, however, there were multiple agencies operating in the ETUs’ districts, and the government-run District Ebola Response Center determined where patients were sent.

A minority of patients, tested in the community or at government-managed holding centers before arriving at the ETU, presented with laboratory-confirmed EVD. Most patients, however, presented to the ETU with 1 or more symptoms consistent with EVD but without laboratory confirmation. Upon arrival, all patients without a previously confirmed test for EVD were brought through triage. In triage, patients were screened by trained ETU clinical staff to ensure that they met the clinical case definition for EVD. IMC created guidelines for this process (see supplementary material) based on WHO and Médecins Sans Frontières (MSF) guidelines and in consultation with local health authorities.^10^^-^^13^ Patients who met the case definition were admitted to the ETU, while those who did not were referred to another operating public or private health care facility, when available, for necessary care.

Patients arriving at an ETU who met the case definition for Ebola virus disease were admitted, while those who did not were referred elsewhere for care.

After triage at the ETU, patients without previously confirmed EVD but who met the case definition were brought to the ward for either suspect or probable disease. There they had a blood sample drawn for initial EVD testing, within 24 hours. Patients with an initial negative test result who had had symptoms for fewer than 3 days were held for repeated testing until 72 hours had passed since the onset of their symptoms. Patients with a second negative test result after having symptoms for more than 3 days were considered EVD-negative (EVD-) and were discharged home from the suspect or probable ward or were transferred to another health care facility for further care as soon as logistically possible. Patients with a positive test result were considered EVD-positive (EVD+) and were moved to the ETU’s confirmed ward for further management, as were patients who presented to the ETU with laboratory-confirmed EVD.

### Laboratory Testing

For both the Bong and Margibi ETUs, laboratory diagnosis of EVD was performed at the United States Naval Medical Research Center (NMRC) Mobile Laboratory in Bong County, Liberia. Diagnosis was confirmed with the 1-step quantitative Ebola Zaire real-time reverse transcriptase–polymerase chain reaction (RT-PCR) (TaqMan) assay (NMRC, Frederick, MD). Briefly, Qiagen Buffer AVL and ethanol-inactivated blood samples were extracted with QIAamp Viral RNA Mini Kit. Extracted ribonucleic acid was tested for 2 EVD gene targets (Zaire ebolavirus [EBOV] locus and minor groove binding locus), using the Applied Biosystems StepOnePlus instrument. A sample was confirmed to be positive for EVD if both targets were detected, but was considered indeterminate if only 1 target was detected. An indeterminate result led to retesting the patient.

In Sierra Leone, the Public Health England (PHE) laboratories in Port Loko and Bombali districts performed EVD testing for patients admitted to the Lunsar and Makeni ETUs, while the Nigerian laboratory in Kambia District (supported by the European Union Mobile Laboratory Consortium) provided RT-PCR testing for patients admitted to the Kambia ETU. The processes were similar to those used by the NMRC laboratory, except that the PHE and Nigerian laboratories tested only a single EVD gene target (EBOV locus) as opposed to 2 targets. In addition, the PHE laboratories switched from using the commercially available Altona real-time RT-PCR assay to using the in-house Trombley assay in February 2015.^14^^-^^16^

The PHE and Nigerian laboratories in Sierra Leone performed malaria tests in addition to EVD tests. However, no other laboratory diagnostics were consistently available for any of the 5 ETUs.

### Clinical Management

All IMC ETU patients were treated according to standard treatment protocols that were based on guidelines developed by WHO and MSF during prior outbreaks and adapted by IMC, in consultation with local Ministry of Health officials, to the needs and resources of Liberia and Sierra Leone.^12^^,^^13^ Briefly, the standard clinical protocol included empiric antimalarial treatment; broad-spectrum antibiotics; oral rehydration solution (ORS); medications to prevent gastritis; vitamins and nutritional supplementation; and symptomatic treatment for fever, pain, nausea, and delirium. Those who presented with or developed moderate to severe dehydration or inability to drink sufficient ORS independently were treated with boluses of crystalloid solution. The standard clinical and psychosocial procedures manual provided with the supplementary materials includes detailed information on all treatments provided.

All patients at IMC ETUs were treated according to standard treatment protocols adapted to the needs of the host country.

Patients were cared for by trained hygienists, nurses, physician assistants, physicians, or psychosocial support staff.

Throughout their inpatient course, patients were cared for by trained hygienists, nurses, physician assistants, physicians, or psychosocial support staff. In general, patients were rounded on 1 to 2 times per day by a physician or physician assistant, who documented clinical signs and prescribed treatments, and 3 to 6 times per day by either a nurse, who provided treatment, or a hygienist, who disinfected the environment to prevent spread of the disease in the wards. Because the suspect, probable, and confirmed wards were all located in the high-risk zone of the ETU, all staff entering this area were required to wear full personal protective equipment (PPE), including scrubs, boots, Tyvek or Tychem suits, masks, hoods, goggles, aprons, and double latex gloves. This limited clinician rounds to 1 to 2 hours at a time due to the heat stress caused by the PPE. The limited rounds meant that all clinical care had to be provided within those periods of rounding, and that for the majority of the day, patients in the wards were unsupervised by clinical staff. At times, however, patients were supported by fellow ETU patients or by EVD survivors who served as caregivers for the very sick.

All staff entering the high-risk patient care areas were required to wear full personal protective equipment.

All forms used in the high-risk zone were considered contaminated, and various methods were used to transfer data out of the ETU.

**Figure:**
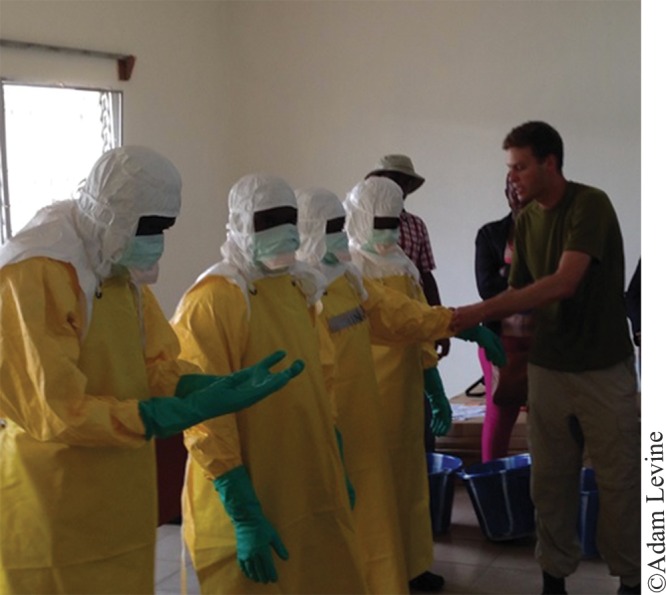
Dr. Adam Levine of IMC’s Ebola research team trains staff at the Bong County Ebola treatment unit in Liberia on how to don personal protective equipment.

### Clinical Documentation and Data Collection

In consultation with local and national ministries of health, WHO, and other international organizations, IMC developed forms to capture demographic, clinical, and psychosocial support data on patients admitted to our ETUs. Examples of these forms, which varied slightly by ETU, are provided as supplementary materials. Triage, laboratory, and discharge forms captured information on patient demographics, presenting symptoms, laboratory test results, and final outcomes and were kept in patient files in the low-risk (nonclinical) area of the ETU. Patient rounding and treatment forms, which were filled out in the high-risk zone of the ETU at the patient’s bedside, included data on patient vital signs and symptoms and on treatments given. Psychosocial support forms included data on mental health symptoms and family and caregiver support, and were filled out once patients were well enough to receive psychosocial support in either the low- or high-risk zone. The data forms were designed to track information on all patients admitted into our ETUs at each critical juncture in their stay from admission through discharge and community follow-up. Forms were also specifically designed with a system of check boxes to minimize the time clinicians spent charting while wearing full PPE.

IMC developed forms designed to track information on all patients admitted to the ETUs, from admission through discharge and community follow-up.

Because all forms in the high-risk zone were considered contaminated, various methods were employed to transfer data from these forms out of the ETU. In some cases, the information was read across the fence between the high- and low-risk zones after rounds were completed each day. In these cases, data was read by one staff member, copied onto an identical chart by another staff member, and then placed in the patient’s file in the low-risk zone. In other cases, charts from the high-risk zone were either imaged from across the fence between high- and low-risk zones or imaged inside the high-risk zone using a waterproof camera. The camera was then decontaminated by soaking in a chlorine solution for 30 minutes before being transferred to the low-risk zone, where the images were downloaded onto a laptop computer. All patient files were eventually scanned into PDF, JPEG, or TIFF format within the low-risk zone of each ETU. All data were collected as part of routine clinical care and for epidemiologic purposes.

Patient data, from paper forms or scanned images, were entered into separate electronic databases at each ETU by local data officers and were later combined into a unified database. The combined database was relational in structure and included 10 separate tables encompassing patient demographic, triage, rounding, treatment, laboratory, psychosocial support, outcome, and follow-up data.

At the conclusion of IMC’s ETU program, scanned images of patients’ paper records were stored in IMC’s secure network drive, and the hard copies were transferred to the Ministry of Health in each country. Camera images from high-risk zones in Liberia were stored in this same IMC network drive. Laboratory data, including EVD RT-PCR cycle thresholds, as well as malaria test results from Sierra Leone, were obtained from the NMRC, PHE, and Nigerian laboratories and linked to patient data in IMC’s unified database.

### Data Quality Audit and Reentry

In November 2015, we used lot quality assurance sampling (LQAS), a random sampling methodology, to assess the quality of the data entered from original patient charts into the ETU-specific databases.^17^^,^^18^ A random sample of 19 patient ID numbers from 2 substrata, EVD+ and EVD-, were selected from each ETU (except Margibi, where 19 total patient ID numbers were randomly selected because only 5 EVD+ patients were admitted) for this data quality audit.

Due to a high number of discrepancies found among triage, rounding, and treatment patient charts and data entered in the unified database, we reentered data using scanned files of original patient charts. Triage data were reentered for all admitted patients; daily rounding and treatment data were reentered only for EVD+ patients, to prioritize limited resources. We took the following steps to ensure minimal errors during data reentry: (1) using data validation settings in Excel reentry documents, (2) using a codebook to ensure that patient data from various types of patient charts were standardized, (3) conducting additional audits by data entry research assistants, and (4) discussing data entry concerns with the principal investigator.

Once reentry was complete, we conducted another data quality audit using LQAS. From each ETU, we selected 19 patient IDs from 2 substrata, EVD+ and EVD- (except in Margibi). We then compared data on scans of EVD+ and EVD- triage, EVD+ rounding, and EVD+ treatment in patient charts with data in the unified database. Each discrepancy was recorded as an error. The number of errors per patient chart was divided by the total number of data points for the specific patient, which depended on the patient’s length of stay. The total percentage of errors was then calculated. With the results from this audit, we concluded that approximately 99% of the data in IMC’s unified database were consistent with information from scans of patient charts. Table 1 summarizes the results of the LQAS.

Steps were taken to ensure data quality, including manually reentering data from scanned files.

**TABLE 1:** Quality of Data Entered From Original Patient Charts Into ETU-Specific Databases: Results of LQAS Audit, Liberia and Sierra Leone, November 2015 (N = 627 Patient Forms)

Patient Form	% Data Entered Correctly
Demographic	99.5%
Triage	99.4%
Rounding	98.1%
Treatment	99.1%
Discharge	99.8%
Overall	99.8%

Abbreviations: ETU, Ebola treatment unit; LQAS, lot quality assurance sampling.

### Data Analysis

The primary outcome variables of interest for patients admitted to the ETUs were final diagnosis (confirmed Ebola, probable Ebola, or other), disposition (survived, deceased, or transferred), and length of stay in the ETU. Length of stay was calculated as the number of days from date of admission to date of discharge, inclusive of the date of admission. Other variables of interest included demographic variables such as country of origin, sex, and age. Age was categorized based on WHO identification of infants under 1 and children under 5 as particularly vulnerable and then into 10-year blocks. We analyzed ETU admission trends by categorizing the date of admission into epidemiological weeks consistent with WHO usage (Monday through Sunday). Clinical variables at triage, including fever, were self- or-family-reported and categorized as yes (1) or no (0).

**Figure:**
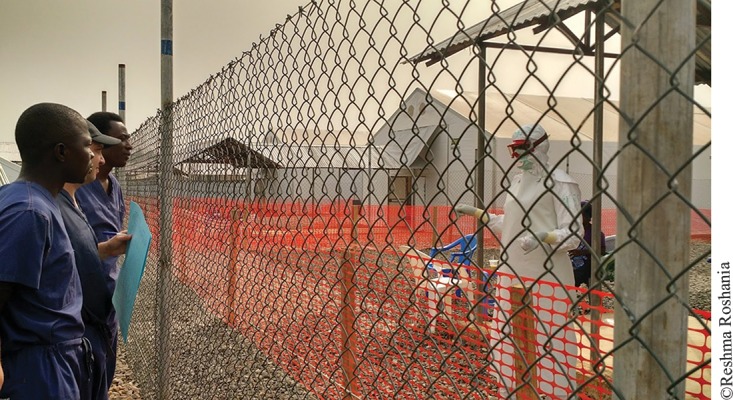
Staff at the Lunsar Ebola treatment unit in Sierra Leone transfer patient data from the high‐risk zone to the low‐risk zone.

We used geographic information system (GIS) software to visualize the geographic distribution of total admitted patients and total EVD+ patients by subregion (e.g., chiefdom or district in which a patient’s home village was located). The maps generated for this analysis display the sum of records per subregion. For the map of total confirmed cases, a defined interval classification method with an interval size of 15 was used to display the number of confirmed Ebola diagnoses across the subregions included in the EVD data set; the map of total patients uses a modified natural breaks (Jenks) classification method. More information about the GIS methods used is provided as supplementary material.

Basic descriptive statistics were calculated for the primary outcome variables as well as for demographic, clinical, epidemiologic, geographic, and time-dependent variables. We used chi-square analysis to compare clinical and epidemiologic variables present on the patient’s arrival against the patient’s final diagnosis. We conducted bivariate logistic regression analyses to examine differences in outcomes by age, sex, and country of origin, presenting odds ratios (ORs) with 95% confidence intervals (CIs). To assess differences in length of stay by subgroup, we conducted independent samples *t* tests and 1-way analysis of variance (ANOVA) tests, as appropriate. Statistical significance was established at .05. Data analyses were conducted in R version 3.2.1 and ArcGIS for Desktop 10.3.1.

## RESULTS

**FIGURE 1.**
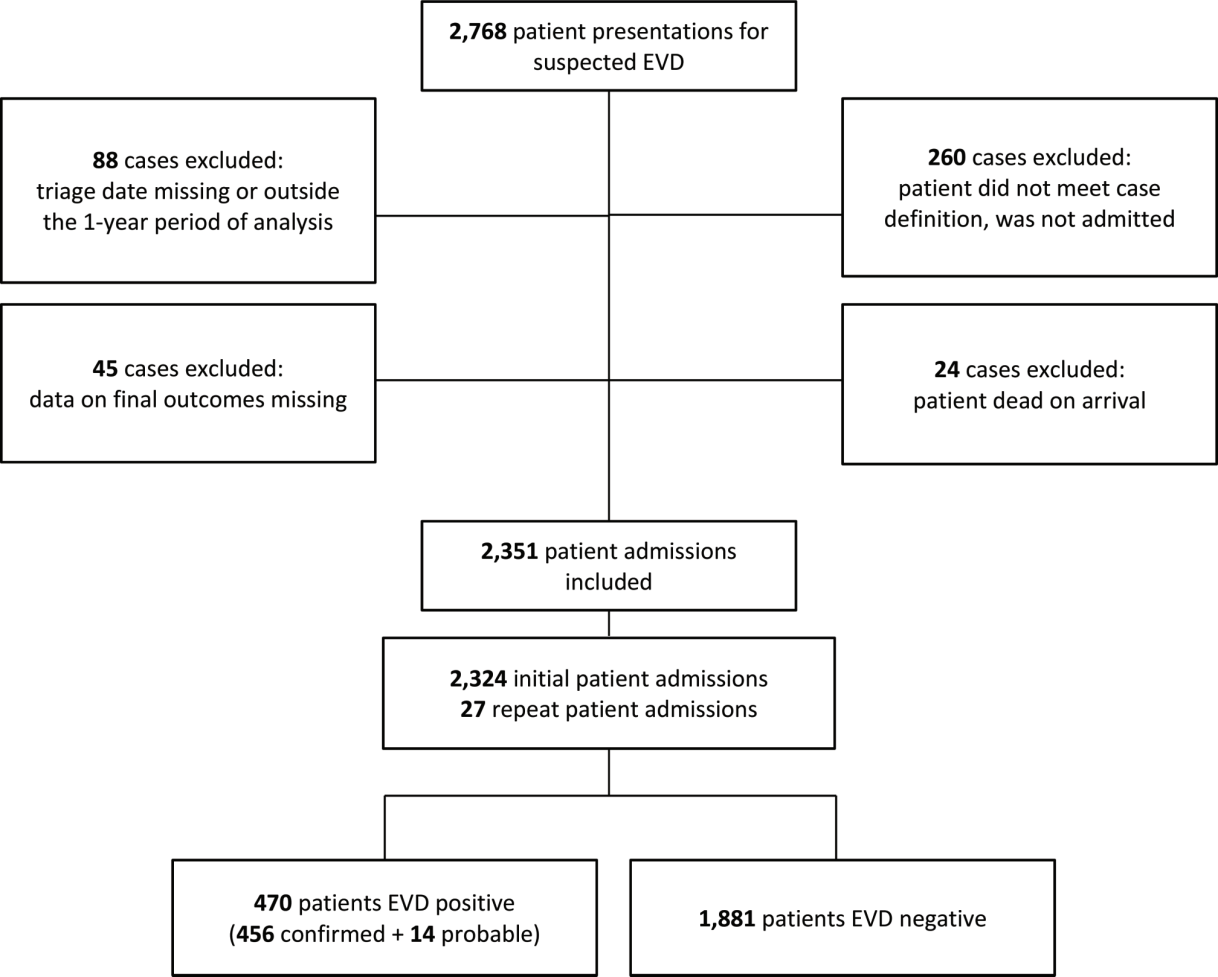
Study Flow Diagram, Liberia and Sierra Leone ETUs, September 15, 2014, to September 15, 2015 Abbreviations: EVD, Ebola virus disease; ETU, Ebola treatment unit.

The full IMC data set contained information on 2,768 total patients presenting to our 5 ETUs. To ensure full follow-up data was available, we excluded from the analysis 88 patients whose data were either missing the date of triage or who were triaged outside the selected 1-year time period. Patients who were declared dead on arrival (n = 24) and those who were not admitted because they did not meet the predefined case definition (n = 260) were also excluded from analysis. Finally, 45 patients with missing data on EVD outcomes (final diagnosis and/or disposition) were also excluded, leaving 2,351 separate patient admissions for analysis (Figure 1).

### Longitudinal and Geographic Data

**FIGURE 2.**
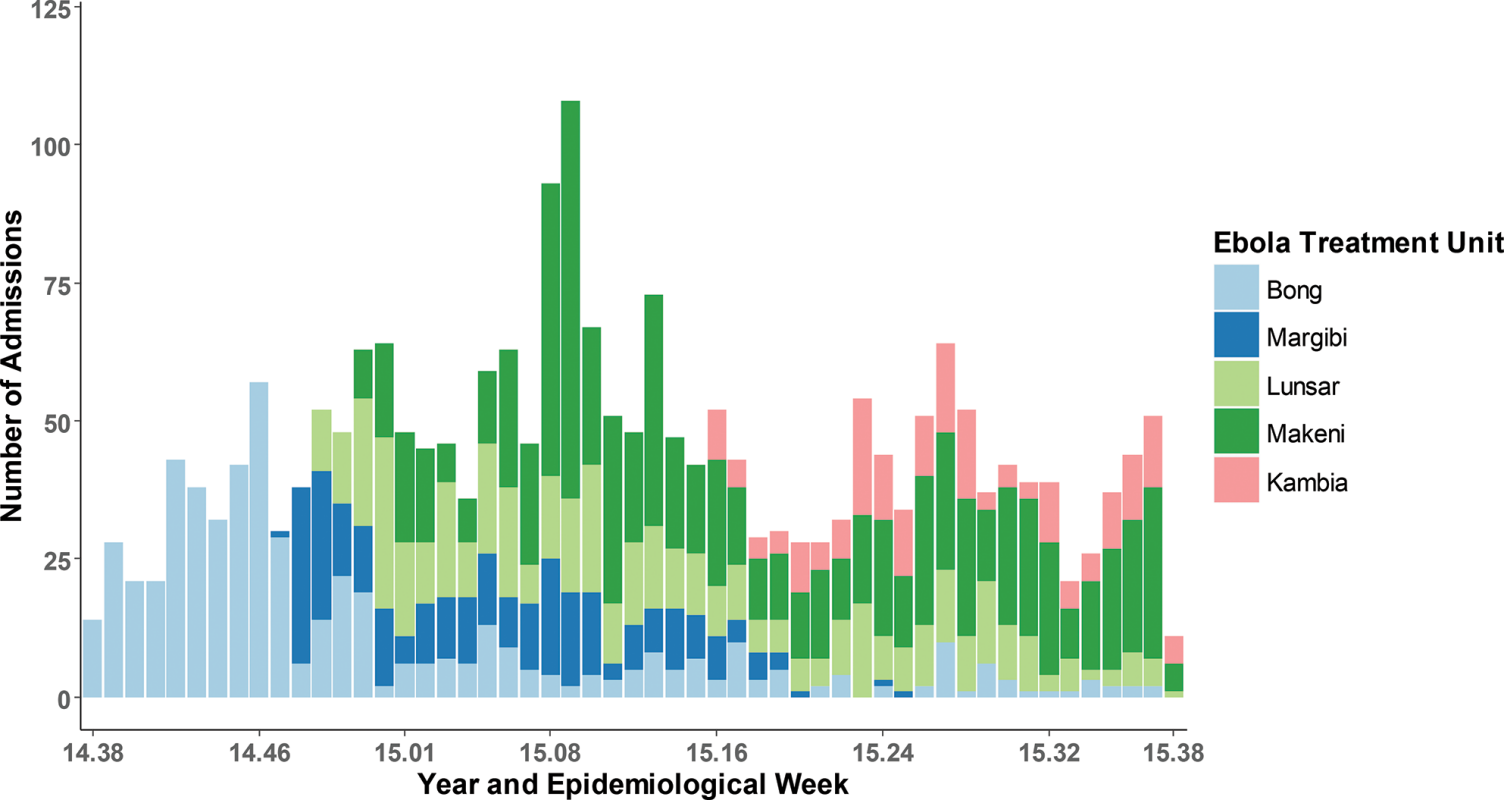
Patient Admissions by ETU^a^ and Epidemiological Week, Liberia and Sierra Leone, September 15, 2014, to September 15, 2015 (N=2,351) Abbreviation: ETU, Ebola treatment unit. ^a^ ETUs were located in Bong County and Margibi County in Liberia, and in Kambia, Lunsar, and Makeni in Sierra Leone.

**FIGURE 3.**
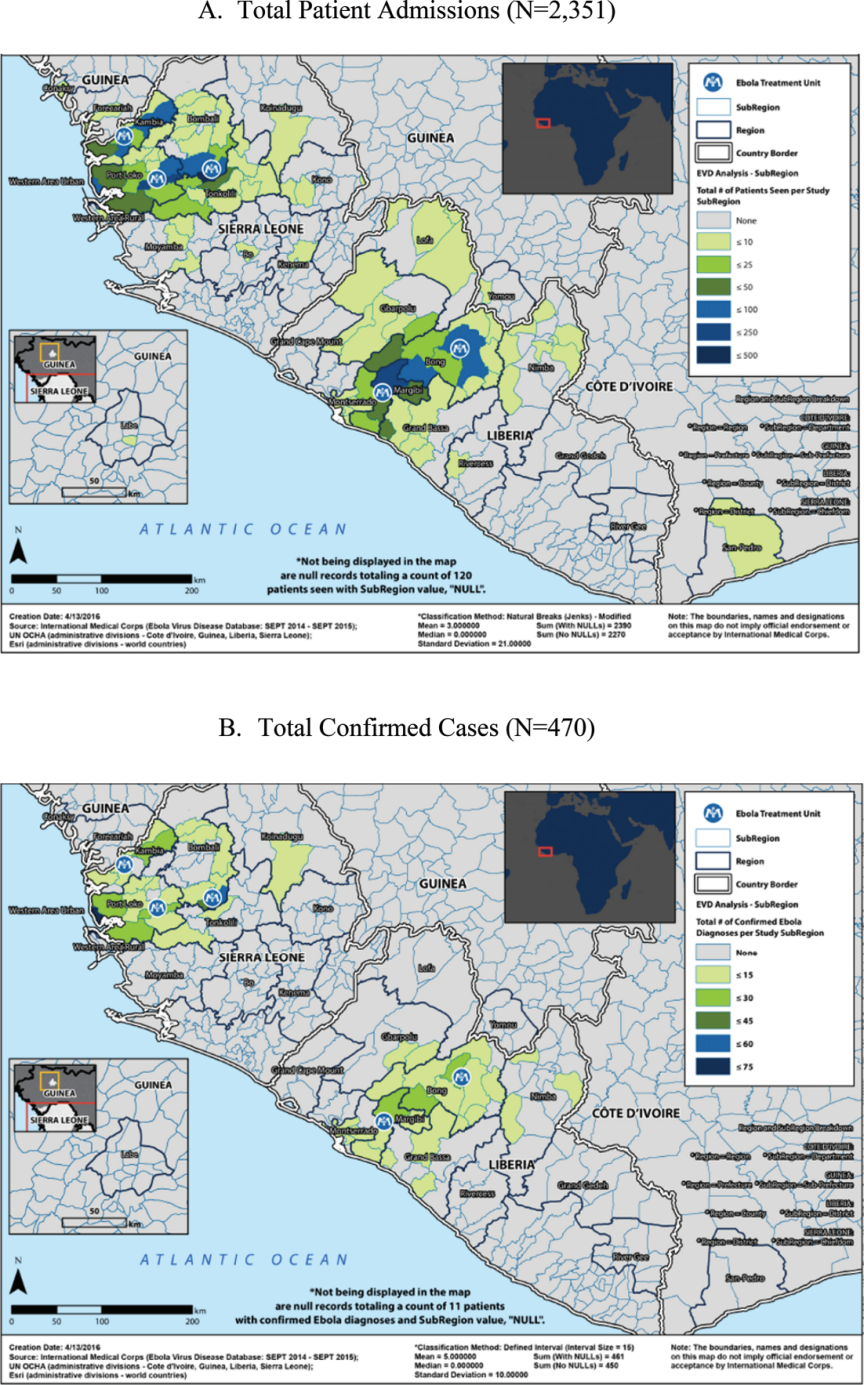
Geographic Distribution of Patients (A) Admitted and (B) Confirmed EVD Positive, Liberia and Sierra Leone, September 15, 2014, to September 15, 2015 Abbreviation: EVD, Ebola virus disease.

Figure 2 shows total patient admissions by ETU by epidemiological week between September 15, 2014, and September 15, 2015. Overall, 1,524 (64.8%) patients were admitted to the 3 ETUs in Sierra Leone, and 827 (45.2%) were admitted to the 2 ETUs in Liberia. Figure 3 shows the distribution of patients’ geographic origin by both total patient admissions (Figure 3A) and confirmed EVD+ patient admissions (Figure 3B). The supplemental figures in the GIS supplementary material provide additional information on the proportions of EVD+ and deceased patients by geographic origin as well as on the numbers of patients EVD+ by the location where they first became ill.

### Demographic Data

Of the 2,351 patient admissions analyzed, 53.4% were men and 46.6% were women. The median age was 30 years (interquartile range: 18, 43). Almost 10% of admitted patients were under age 5, and 11% were 55 years or older, while nearly 25% were 25 to 34 years old (Table 2).

**TABLE 2:** Outcomes Among All Admitted Patients by EVD Status, Sex, Age, and Location, Liberia and Sierra Leone, September 15, 2014, to September 15, 2015

	EVD+ Recovered	EVD+ Deceased^a^	EVD+ Transferred	EVD+ Total	EVD- Discharged	EVD- Deceased	EVD- Total	All Patients
All patients	197 (8.4)	268 (11.4)	5 (0.2)	470 (20.0)	1,725 (73.4)	156 (6.6)	1,881 (80)	2,351 (100)
Sex^b^								
Men	76 (6.1)	107 (8.5)	3 (0.2)	190 (15.2)	959 (76.6)	97 (7.7)	1,062 (84.8)	1,252 (53.4)
Women	118 (10.8)	159 (14.6)	2 (0.2)	279 (25.6)	754 (69.1)	58 (5.3)	812 (74.4)	1,091 (46.6)
Age, years^b^								
0 to < 1	1 (1.9)	13 (24.5)	0 (0.0)	14 (26.4)	36 (67.9)	3 (5.7)	39 (73.6)	53 (2.3)
1 to 4	4 (2.4)	28 (16.5)	0 (0.0)	32 (18.8)	127 (74.7)	11 (6.5)	138 (81.2)	170 (7.3)
5 to 14	36 (15.5)	29 (12.4)	1 (0.4)	66 (28.3)	158 (67.8)	9 (3.9)	167 (71.7)	233 (10.0)
15 to 24	44 (10.8)	27 (6.7)	1 (0.2)	72 (17.7)	312 (76.8)	22 (5.4)	334 (82.3)	406 (17.4)
25 to 34	45 (8.1)	42 (7.6)	2 (0.4)	89 (16.1)	434 (78.5)	30 (5.4)	464 (83.9)	553 (23.6)
35 to 44	33 (8.8)	53 (14.2)	1 (0.3)	87 (23.3)	260 (69.5)	27 (7.2)	287 (76.7)	374 (16.0)
45 to 54	17 (5.8)	36 (12.3)	0 (0.0)	53 (18.1)	214 (73.0)	26 (8.9)	240 (81.9)	293 (12.5)
≥ 55	16 (6.2)	40 (15.5)	0 (0.0)	56 (21.7)	175 (67.8)	27 (10.5)	202 (78.3)	258 (11.0)
Country								
Sierra Leone	114 (7.5)	173 (11.4)	5 (0.3)	292 (19.2)	1,122 (73.6)	110 (7.2)	1,232 (80.8)	1,524 (64.8)
Liberia	83 (10.0)	95 (11.5)	0 (0.0)	178 (21.5)	603 (72.9)	46 (5.6)	649 (78.5)	827 (35.2)

Abbreviation: EVD, Ebola virus disease.

All data reported as No. (%).

a Patients with suspected EVD (n = 14) included in EVD+ deceased.

b Missing values not included.

### Clinical and Epidemiologic Data

Table 3 lists the clinical symptoms and epidemiologic characteristics of patients admitted to the 5 ETUs for triage, stratified by their final diagnosis. Clinical symptoms, including fever, were self- or family-reported and endorsed. While fever (75.3%, *P* = .11), weakness (71.9%, *P* = .76), and loss of appetite (68.4%, *P* = .20) were the most common symptoms in patients with EVD, they were equally common in patients without EVD. Among clinical symptoms, only diarrhea (54.0%, *P*<.001) and red eyes (27.5%, *P*<.001; a variable that included both conjunctivitis and conjunctival hemorrhage) were more common in patients with EVD than in those with other diagnoses. Abdominal pain (54.0% vs. 43.5%, *P*<.001), shortness of breath (30.9% vs. 23.5%, *P* = .002), and non-ocular bleeding (11.3% vs. 5.5%, *P*<.001) were actually more common at triage among patients without EVD than those with EVD.

At triage, diarrhea and red eyes were more common among patients diagnosed with EVD than those without. Abdominal pain, shortness of breath, and non-ocular bleeding were more common among patients without EVD.

**TABLE 3:** Chi-Square Analysis of Symptoms Reported at Triage by Patients With and Without EVD, Liberia and Sierra Leone, September 15, 2014, to September 15, 2014

	EVD+ Patients, No (%)	EVD- Patients, No. (%)	*P* Value
Clinical symptom			
Fever	353 (75.3)	1,481 (78.7)	.11
Asthenia (weakness)	337 (71.9)	1,338 (71.1)	.76
Loss of appetite	321 (68.4)	1,228 (65.3)	.20
Headache	273 (58.2)	1,109 (59.0)	.77
Myalgia or arthralgia (muscle or joint pain)	273 (58.2)	1,093 (58.1)	.97
Nausea or vomiting	225 (58.0)	947 (60.4)	.38
Diarrhea	235 (54.0)	662 (37.4)	< .001
Abdominal pain	204 (43.5)	1,016 (54.0)	< .001
Red eyes^a^	129 (27.5)	189 (10.1)	< .001
Sore throat or difficulty swallowing	112 (23.9)	440 (23.4)	.82
Dyspnea (shortness of breath)	110 (23.5)	581 (30.9)	.002
Hiccups	57 (12.2)	246 (13.1)	.59
Jaundice	24 (5.1)	108 (5.7)	.60
Bleeding, non-ocular	26 (5.5)	212 (11.3)	< .001
Epidemiologic variable			
Had contact with someone ill	340 (82.1)	367 (21.5)	< .001
Attended funeral	144 (39.7)	146 (8.8)	< .001
Had recent travel outside of home district	18 (11.7)	122 (20.0)	.02
Worked in health sector	9 (4.2)	62 (6.5)	.20
Had contact with bush meat	0 (0.0)	13 (2.5)	.06

Abbreviation: EVD, Ebola virus disease.

a Includes red injected eyes, conjunctivitis, and hemorrhagic eyes.

Among epidemiologic variables, any contact with a sick person (82.1% vs. 21.5%, *P*<.001) and attendance at a funeral (39.7% vs. 8.8%, *P*<.001) were far more common among patients with EVD. Eating bush meat and working in health care were both uncommon at triage and were not associated with a final EVD diagnosis, while recent travel was somewhat more likely in patients without EVD.

20% of the patients admitted to IMC ETUs for triage tested positive for EVD.

### Outcome Data

**FIGURE 4.**
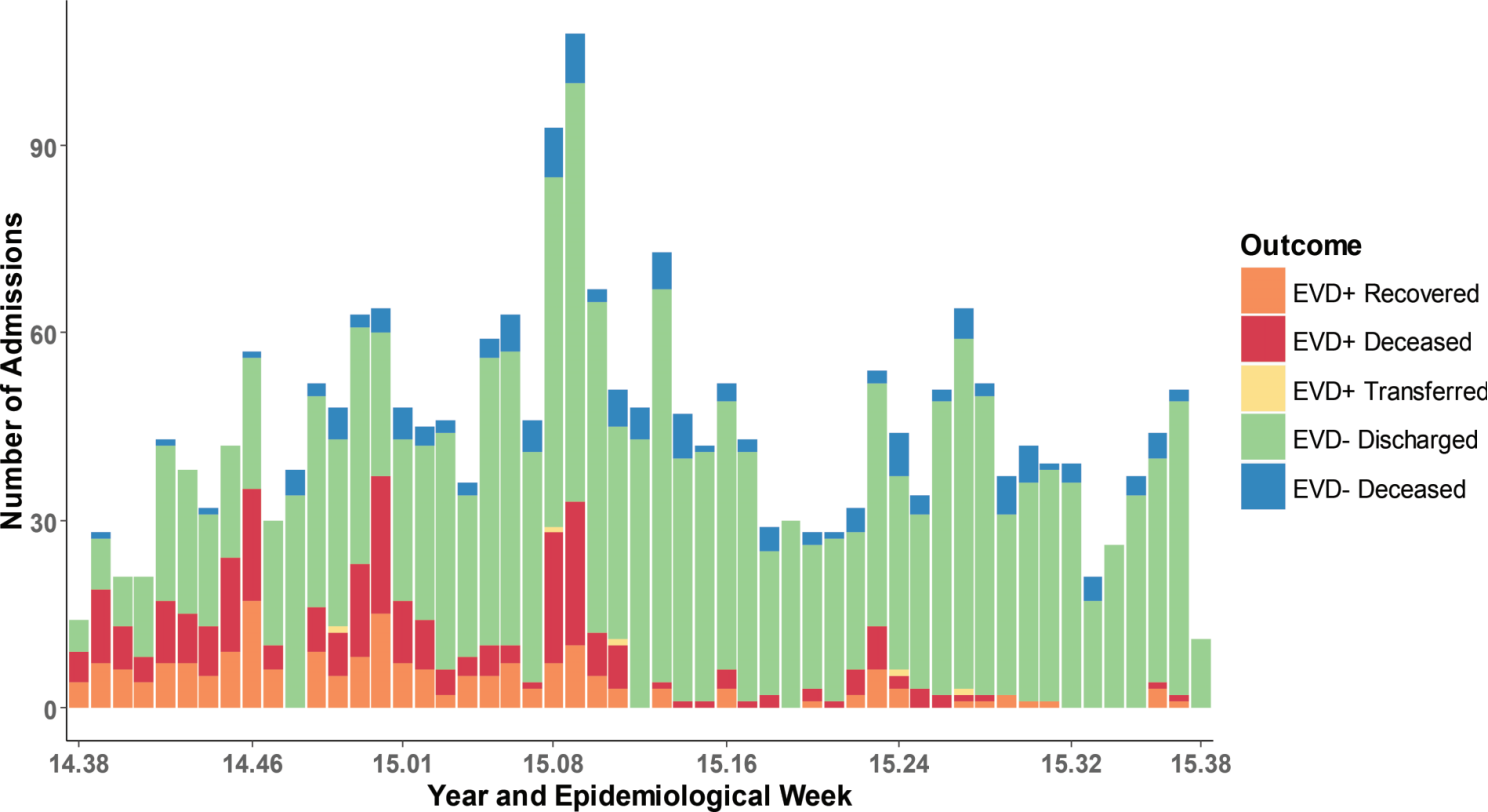
Final Diagnosis and Outcome of Admitted Patients by Epidemiological Week, Liberia and Sierra Leone, September 15, 2014, to September 15, 2015 (N=2,351) Abbreviation: EVD, Ebola virus disease.

Among all patients admitted for triage, 470 (20%) tested positive for EVD, including 14 patients with probable EVD who died before laboratory testing. Of these 470 patients, 197 recovered to discharge, 5 were transferred to other facilities (final outcome unknown), and 268 died. The overall case fatality ratio (CFR) was 57%. Among EVD- patients, 156 of 1,881 admissions also died during their stay in the ETU, for an overall CFR of 8.1%. Figure 4 shows the total number of patients admitted per week across all 5 ETUs, by diagnosis and outcome.

Average length of stay was 4.7 (standard deviation [SD] = 3.9) days for all admitted patients, although this differed greatly based on diagnosis and outcome (Table 4). The average length of stay for EVD+ patients who recovered was 14.7 (SD = 5.5) days; the average for EVD+ patients who died was 5.6 (SD = 3.2) days (*P*<.001). For EVD- patients, average length of stay was 3.6 (SD = 1.6) days for those who survived and 2.3 (SD = 1.4) days for those who died (*P*<.001).

**TABLE 4:** Average Length of Stay (in Days) in ETU by EVD Status, Outcome, Sex, Age, and Location, Liberia and Sierra Leone, September 15, 2014, to September 15, 2015

	EVD+^a^				EVD-				All Patients	
	Recovered		Deceased^b^		Discharged		Deceased			
	n	ALOS	n	ALOS	n	ALOS	n	ALOS	n	ALOS
All patients	197	14.7	268	5.6	1,721	3.6	156	2.3	2,346	4.7
Sex^c^										
Men	77	13.6	109	5.6	963	3.6	98	2.2	1,250	4.2
Women	118	15.4	159	5.6	751	3.7	58	2.4	1,088	5.2
Age, years^c^										
0 to < 1	1	18.0	13	6.0	36	4.2	3	1.7	53	4.8
1 to 4	4	23.0	28	6.6	127	3.8	11	1.9	170	4.6
5 to 14	36	15.4	29	5.5	158	3.6	9	3.2	233	5.7
15 to 24	44	14.4	27	4.7	311	3.5	22	2.0	405	4.7
25 to 34	44	13.5	42	5.8	433	3.5	30	2.6	551	4.4
35 to 44	33	15.0	53	5.4	260	3.6	27	1.9	374	4.8
45 to 54	17	13.4	36	5.8	213	3.4	26	2.1	292	4.2
≥ 55	16	15.3	40	5.3	174	3.9	27	2.4	257	4.7
Country										
Sierra Leone	113	14.6	173	5.6	1,119	4.0	110	2.4	1,520	4.9
Liberia	83	14.9	95	5.7	602	2.9	46	2.0	826	4.3

Abbreviations: ALOS, average length of stay; ETU, Ebola treatment unit; EVD, Ebola virus disease.

a EVD+ patients who were transferred (n = 5) not included.

b Patients with suspected EVD (n = 14) included in EVD+ deceased.

c Missing values not included.

### Outcome by Age, Sex, and Location

More men than women were admitted to IMC ETUs. However, a greater proportion of the women admitted were diagnosed with EVD.

**FIGURE 5.**
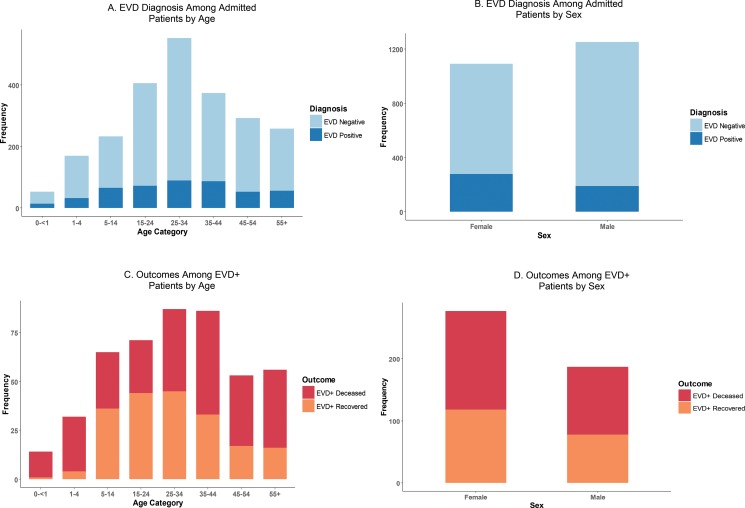
Final Diagnosis Among Admitted Patients (N=2,351) and Mortality Outcomes Among EVD+ Patients (N=465) by Age and Sex, Liberia and Sierra Leone, September 15, 2014, to September 15, 2015 Abbreviation: EVD, Ebola virus disease.

Figure 5A and Figure 5B show the proportion of patients diagnosed with EVD by age and by sex. Although a higher absolute number of patients admitted to the 5 ETUs were men (53.4% vs. 46.6%; see Table 2), a much larger proportion of women admitted were actually diagnosed with EVD (25.6% vs. 15.2%, *P*<.001). Figure 5C and Figure 5D show outcomes for EVD+ patients (i.e., deceased or recovered) by age and sex.

Table 5 summarizes the CFR among EVD+ patients by age, sex, and location. As shown in Figure 5 and Table 5, EVD+ patients ages 15 to 24 had the lowest CFR (38.0%), while the youngest and oldest patients had the highest CFRs (89.1% for patients under 5; 71.4% for patients over 55). As compared with patients aged 15 to 24, children under 5 had a mortality odds ratio of 12.8 (95% CI, 4.8 to 41.3; *P*<.001), while patients over 55 had a mortality odds ratio of 4.0 (95% CI, 1.9 to 8.7; *P*<.001). No significant differences were found among EVD+ patients in the odds of death based on sex (OR = 1.0l; 95% CI, 0.7 to 1.4; *P* = .09) or country of origin (OR = 0.8; 95% CI, 0.5 to 1.1; *P* = .15).

**TABLE 5:** Case Fatality Ratios Among Patients With EVD by Gender, Age, and Location, September 15, 2014, to September 15, 2014

	EVD+ Recovered, No. (%)	EVD+ Deceased,^a^No. (%)	Total,^b^No. (%)	EVD Fatality,OR (95% CI)	*P* Value
All patients	197 (42.4)	268 (57.6)	465 (100)		
Sex^c^					
Men	78 (41.7)	109 (58.3)	187 (40.3)	-	-
Women	118 (42.6)	159 (57.4)	277 (59.7)	1.0 (0.7, 1.4)	.09
Age, years^c^					
0 to <1	1 (7.1)	13 (92.9)	14 (3.1)	18.3 (3.3, 463.5)	< .001
1 to 4	4 (12.5)	28 (87.5)	32 (7.0)	10.8 (3.7, 40.8)	< .001
5 to 14	36 (55.4)	29 (44.6)	65 (14.0)	1.3 (0.7, 2.6)	.49
15 to 24	44 (62.0)	27 (38.0)	71 (15.3)	-	-
25 to 34	45 (51.7)	42 (48.3)	87 (18.8)	1.5 (0.8, 2.9)	.20
35 to 44	33 (38.4)	53 (61.6)	86 (18.5)	2.6 (1.4, 5.0)	.004
45 to 54	17 (32.1)	36 (67.9)	53 (11.4)	3.4 (1.6, 7.4)	.001
≥ 55	16 (28.6)	40 (71.4)	56 (12.1)	4.0 (1.9, 8.7)	< .001
Country					
Sierra Leone	114 (39.7)	173 (60.3)	287 (61.7)	-	-
Liberia	83 (46.6)	95 (53.4)	178 (38.3)	0.8 (0.5, 1.1)	.15

Abbreviations: CI, confidence interval; EVD, Ebola virus disease; OR, odds ratio.

a Patients with suspected EVD (n = 14) included in EVD+ deceased.

b EVD+ patients who were transferred (n = 5) not included.

c Missing values not included.

## DISCUSSION

With nearly 30,000 cases of EVD, the 2014 outbreak in West Africa dwarfed all prior epidemics of viral hemorrhagic fever.^1^ At its height, this Ebola epidemic completely overwhelmed the limited response capacity of the 3 most-affected countries and led to an untold number of secondary deaths due to breakdowns in the local health care and public health systems.^19^^,^^20^ However, as horrific as this epidemic has been for the people of West Africa, it likely could have been far worse had the international community failed entirely to respond. Recent mathematical modeling from Sierra Leone suggests that the introduction of treatment beds for patients with EVD between June 2014 and February 2015 averted an estimated 56,600 cases, while opening ETUs just 1 month earlier could have averted an additional 12,500 cases.^21^

Among patients with EVD, those ages 15 to 24 had the lowest case fatality rate; the youngest and oldest patients had the highest case fatality rates.

IMC, in collaboration with local ministries of health, contributed to the 2014 Ebola response in West Africa by opening 5 ETUs to manage suspected and confirmed Ebola cases. IMC trained health workers in infection prevention and control, EVD clinical management, and ETU operations and worked in communities to build awareness around Ebola response activities. While the efforts of IMC and other humanitarian organizations to provide direct medical care to patients and limit the spread of the epidemic has been well recorded by the lay media, another critical activity IMC engaged in has gone almost completely unnoticed: Staff at IMC ETUs collected clinical and epidemiologic data in the most austere and difficult of circumstances.^22^^,^^23^

When IMC first launched its Ebola response in Liberia in September of 2014, very little was known about optimal prevention, treatment, or management strategies for a large-scale EVD outbreak. Despite more than 2 dozen prior outbreaks over the past 4 decades, little empirical evidence existed to guide response operations at the start of this outbreak.^22^ While recent reports have documented the experiences of individual ETUs in Guinea and Sierra Leone, no prior studies have presented data from multiple ETUs across multiple countries run by the same organization with similar clinical protocols.^5^^-^^9^ The lack of empiric evidence made it difficult to develop standardized clinical protocols for patient care. More importantly, the lack of evidence made it nearly impossible to prioritize our limited available resources for those who might benefit the most, especially early in the response.

As a result of the data collected by our teams in Liberia and Sierra Leone, we know far more about this disease now than when we began our 2014 response. We now have a better understanding of the demographics of patients affected, the expected length of stay in an ETU based on patient diagnosis and outcome, and the expected mortality, for patients both with and without EVD, in a resource-limited environment. We found, for instance, that although fewer women will visit an ETU, a larger proportion of them will have EVD. Where prior publications generally stratified patients into 2 age groupings (young vs. old), we chose to look at patients in 10-year blocks to get a more granular understanding of the effect of age on diagnosis of EVD and patient mortality. This more granular presentation of age revealed some interesting nuances, such as highlighting the age groups with the lowest CFR (15–24 years) and the highest mortality (children under 5 and adults over 55). Our data confirmed that high-risk epidemiological factors for contracting EVD include contact with a sick person and attendance at a funeral, but found that eating bush meat was not a significant risk factor for EVD, and that recent travel was more likely in patients without EVD.

Our data confirmed that high-risk factors for contracting EVD include contact with a sick person and attendance at a funeral.

In addition, prior analyses of our data set have helped us to develop better tools for EVD screening.^24^ We have planned further analyses of our data set as well, which we expect will continue to help us learn about the natural history of this disease and the best ways to diagnose and manage it in resource-limited settings. Our experience in West Africa, however, also taught us important lessons about the many challenges to collecting high-quality data during an epidemic, and the various ways in which these challenges can be overcome.

### Major Challenges to Data Collection and Lessons Learned

#### Lack of Data Standardization

One of the greatest challenges in building our data was the lack of standardization in the data collected across different countries and different ETUs. Despite being managed by the same organization, the various ETUs collected different types of clinical and epidemiologic data in somewhat different formats, and in some cases the types of data collected changed over time. This was due to a variety of factors, including the need to comply with local guidelines and use government-approved triage forms; the emergent nature of the epidemic and the lack of time to agree upon and disseminate standardized data collection forms; and the lack of prior empiric evidence on which data elements were most important to collect in the context of EVD.

Some variables, such as eating bush meat or recent travel, were collected in only 1 of the 2 countries. In some cases, subtle differences between the types of clinical variables collected made it difficult to compare data. For example, we had to group “red injected eyes,” “conjunctivitis,” and “hemorrhagic eyes” into a single variable (“red eyes”) for analysis. In the future, standardized data forms with clear, consensus-based definitions of clinical and epidemiologic variables should be developed and adopted in advance by the international community to support data collection during outbreaks of viral hemorrhagic fever and other diseases of epidemic potential. Such standardized forms will allow for more consistent and comparable data. Furthermore, clinical staff working with these clinical variables should receive training to ensure that knowledge of these variables is properly used in the field. Evidence collected during this epidemic by our organization and others on the symptoms, signs, and tests that are most predictive of EVD diagnosis and outcomes will likely be helpful in developing these new tools; in the end it will require substantial coordination by the international humanitarian community and local governments to put these tools into practice.

**Figure:**
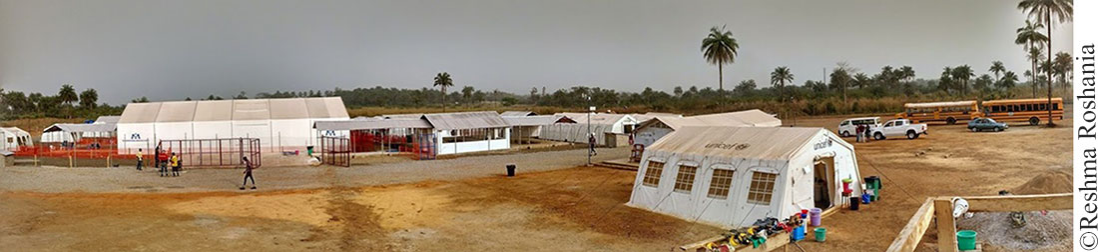
An aerial view of the Lunsar Ebola treatment unit in Sierra Leone.

Standardized data collection forms should be developed and adopted in advance of disease outbreaks.

#### Logistical Constraints

The severe logistical constraints related to collecting data in the setting of a highly contagious and virulent disease such as Ebola cannot be overemphasized. Aside from the initial triage data, which we collected by asking patients or family members questions from across a barrier fence at a distance of 2 meters, all of the daily symptom data and treatment information had to be collected at the patient’s bedside in the ETU’s high-risk zone. The providers collecting the data were dressed in full PPE, which limited both their movements and the time they could spend at the bedside. Clinical data forms thus had to be simple to fill out; it was easiest to use mostly check boxes or circles. Moreover, the paper forms that the data was collected on could themselves easily become contaminated with highly infectious body fluids, making each form a biohazard that could not safely be removed from the high-risk zone. Because clinicians washed their (gloved) hands with chlorine in between each patient, but did not dry their hands, the paper forms also became degraded by chlorine over time. Extraction of the data from these contaminated and degraded forms required either the laborious and error-prone process of reading the data over the fence between risk zones and copying it onto new forms after each set of rounds, attempting to image the forms from across the fence, or sending in additional personnel in full PPE for the sole purpose of imaging the forms with a submersible camera that could be decontaminated and removed from the high-risk zone. While each of these methods were used at different ETUs, no method was ideal.

In the future, other solutions could be considered for more efficient means of collecting data. Electronic medical records, accessed through handheld tablets kept in the high-risk zone of the ETU, might offer an easier way to collect data. Electronic data could then be transmitted via Wi-Fi to a computer located outside of the high-risk zone. Yet while ideal from a data collection standpoint, this method would present challenges in very resource-limited settings: it would be necessary to get the tablets prepositioned and configured before the start of an outbreak, and they would need a working Wi-Fi network. Another option would be a dual system, using paper charts that could be scanned and downloaded onto a laptop within the high-risk zone after each set of rounds, and then electronically transmitted via Wi-Fi when available or even via a simple Ethernet cable to another computer in the low-risk zone.

In the future, additional solutions for collecting data in high-risk zones safely and efficiently should be considered, such as the use of electronic medical records.

#### Data Entry Constraints

The final challenge involved entering data from paper charts or scanned images into an electronic format that could be analyzed. While local data officers entered data into electronic databases in real time at each of the 5 ETUs, the data entry methods and quality control varied by ETU and over time depending on the severity of the epidemic (meaning more patients and more data to enter) and availability of staff. This led to slight differences in the way variables were coded and the formats in which they were coded. Just as the data forms used varied from ETU to ETU, the database software also varied, with some ETUs using Microsoft Access and others using Microsoft Excel. This led to substantial challenges and a great deal of extra work to combine the different data sets. Moreover, our initial quality assurance check demonstrated unacceptably high error rates in some of the data, and it had to be reentered from scanned images of the patient charts. Although we were eventually able to ensure a low error rate of about 1% for our data set overall, much time and effort could have been saved through closer oversight and real-time quality assurance assessments in the field. In addition, just as standardized data forms would have helped, a standardized database with data entry controls (such as preset ranges for certain variables and drop-down lists for other variables, with limited ability to enter free-form text), coupled perhaps with a standardized training for data entry officers, would have simplified the process greatly and led to less data errors in the field.

Time and effort could be saved with standardized data forms, standardized databases, and closer oversight and quality assurance while data are being collected in the field.

## CONCLUSION

Despite the challenges faced, IMC was able to collect roughly 25,000 pages of clinical and epidemiologic data on more than 2,500 patients in the midst of the largest epidemic of viral hemorrhagic fever to date. This data is already helping to improve our operational guidelines and preparedness for the next major outbreak and could help a national Ministry of Health launch a response to a future Ebola outbreak. Hopefully the lessons learned from this experience can also lead to higher-quality and better-coordinated data collection in future epidemics, leading in turn to an improved overall humanitarian response.

## Supplementary Material

supplementary material

## Notes

**Peer Reviewed**
