# Supplementary material for: Successful Implementation of a Multicountry Clinical Surveillance and Data Collection System for Ebola Virus Disease in West Africa: Findings and Lessons Learned
Source: Glob Health Sci Pract. 2016 Sep 28;4(3):394–409. doi: 10.9745/GHSP-D-16-00186 (PMC5042696; doi:10.9745/GHSP-D-16-00186)
Supplement: supplementary material [file GHSP-D-16-00186_index.html]

Supplement to Successful Implementation of a Multicountry Clinical Surveillance and Data Collection System for Ebola Virus Disease in West Africa: Findings and Lessons Learned | Global Health: Science and Practice

## GHSP-D-16-00186 Supplementary Material

Levine et al. doi: 10.9745/GHSP-D-16-00186

- Supplementary Material - Levine et al. doi: 10.9745/GHSP-D-16-00186
- Supplementary Material - Levine et al. doi: 10.9745/GHSP-D-16-00186
- Supplementary Material - Levine et al. doi: 10.9745/GHSP-D-16-00186
