# Supplementary material for: Successful Implementation of a Multicountry Clinical Surveillance and Data Collection System for Ebola Virus Disease in West Africa: Findings and Lessons Learned
Source: Glob Health Sci Pract. 2016 Sep 28;4(3):394–409. doi: 10.9745/GHSP-D-16-00186 (PMC5042696; doi:10.9745/GHSP-D-16-00186)

Roshania R, Mallow M, Dunbar N, Mansary D, Shetty P, Lyon T, et al. Successful implementation of a multicountry clinical surveillance and data collection system for Ebola virus disease in West Africa: findings and lessons learned. Glob Health Sci Pract. 2016;4(3). http://dx.doi.org/10.9745/GHSP-D-16-00186

SUPPLEMENTARY MATERIAL: GIS Analysis

Methods

Beginning the geospatial analysis of the International Medical Corps Ebola virus disease (EVD) data set, fields containing administrative division names per level (1 through 3) were added to the original data set based on the existing columns, *PatientAddressRegion* and *PatientAddressSubRegion*. *PatientAddressRegion* and *PatientAddressSubRegion* represent the region and subregion respectively of the patient's home address.

For geospatial purposes, it was determined that the data would be aggregated according to Region and SubRegion. **Region** would consist of Liberia's administrative level 1 divisions (County) while Sierra Leone, Guinea, and Côte d'Ivoire would each use the corresponding administrative level 2 division data set (District, Prefecture, and Region, respectively). **SubRegion** would then comprise administrative level 2 divisions for Liberia (District) and administrative level 3 divisions for Sierra Leone, Guinea, and Côte d'Ivoire (Chiefdom, Subprefecture, and Department, respectively). The regions and subregions were mapped to differing administrative divisions in this manner to ensure that the subregions mapped were as uniform in size as possible in order to decrease the spatial bias of the modifiable areal unit problem (MAUP).

Using the EVD data set with the new columns of *Region* and *SubRegion*, two summary tables aggregating the original data by Patient Home Address *SubRegion* were created in Excel per requested field-value combination (*Final Diagnosis = Confirmed Ebola* and *Disposition = ALL*) to obtain numeric values per subregion detailing total confirmed EVD cases and total patients seen, respectively. The summary tables were then aggregated into a singular table containing all subregions within the EVD data set and joined to a merged GIS layer encompassing all aforementioned administrative divisions and levels for visualization. The home address region and subregion were then mapped by Confirmed Ebola, Deceased, and both Confirmed and Deceased (Figure 3A and Figure 3B in the main text, and Supplemental Figure 3.1, Supplemental Figure 3.2, and Supplemental Figure 3.3 in this appendix).

The attributes *PatientIllRegion* and *PatientIllSubRegion* were also mapped for this study (Supplemental Figure 3.4). In contrast to *PatientAddressRegion* and *PatientAddressSubRegion*, the attributes *PatientIllRegion* and *PatientIllSubRegion* represent the region and subregion where the patient first became ill. For the initial analysis of patient illness subregion, the data was constrained to *Final Diagnosis = Confirmed Ebola.* A summary table of the data was created which tallied the total number of patients per illness subregion. The summary table was joined to a merged GIS layer encompassing all administrative divisions and visualized in a map (Supplemental Figure 3.4).

The second analysis of patient illness subregion consisted of a comparison of the illness subregion for each patient versus the patient's home address subregion. For each patient ID, the home address subregion (*PatientAddressSubRegion*) was matched to illness subregion (*PatientIllSubRegion*) in Excel using an IF statement which returned TRUE if the locations matched and FALSE if the locations did not match. Then, the data was constrained further to only the confirmed EVD records which had a *PatientAddressSubRegion* that did not match *PatientIllSubRegion.* A summary table of the data was created which tallied the total number of patients per illness subregion where illness subregion did not equal home address subregion. The summary table was then joined to a merged GIS layer of administrative divisions for mapping purposes. Pie charts were also created for each illness subregion (*PatientIllSubRegion*). The pie charts show the proportion of the patients' home address subregions per subregion where they fell ill. (Supplemental Figure 3.5).

Roshania R, Mallow M, Dunbar N, Mansary D, Shetty P, Lyon T, et al. Successful implementation of a multicountry clinical surveillance and data collection system for Ebola virus disease in West Africa: findings and lessons learned. Glob Health Sci Pract. 2016;4(3). http://dx.doi.org/10.9745/GHSP-D-16-00186

Supplemental Figures

SUPPLEMENTAL FIGURE 3.1. Proportion of ETU Admissions Found to be EVD Positive by Patient Home Address, September 15, 2014, to September 15, 2015

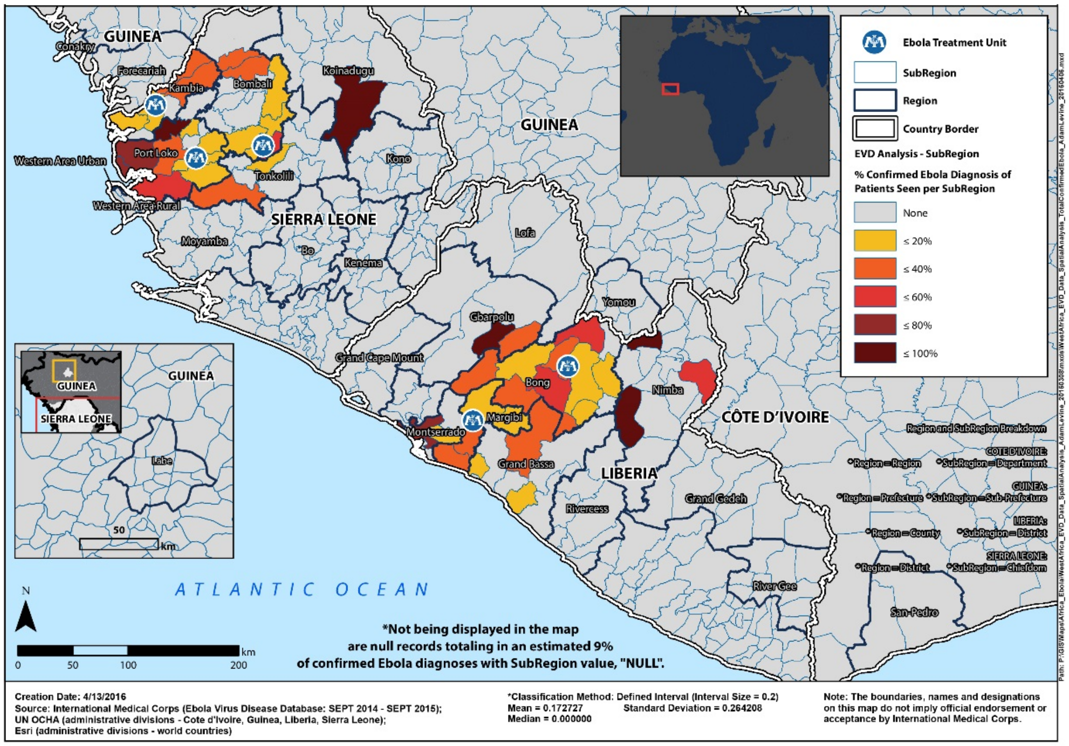

SUPPLEMENTAL FIGURE 3.2. Proportion of ETU Admissions Who Died by Patient Home Address, September 15, 2014, to September 15, 2015

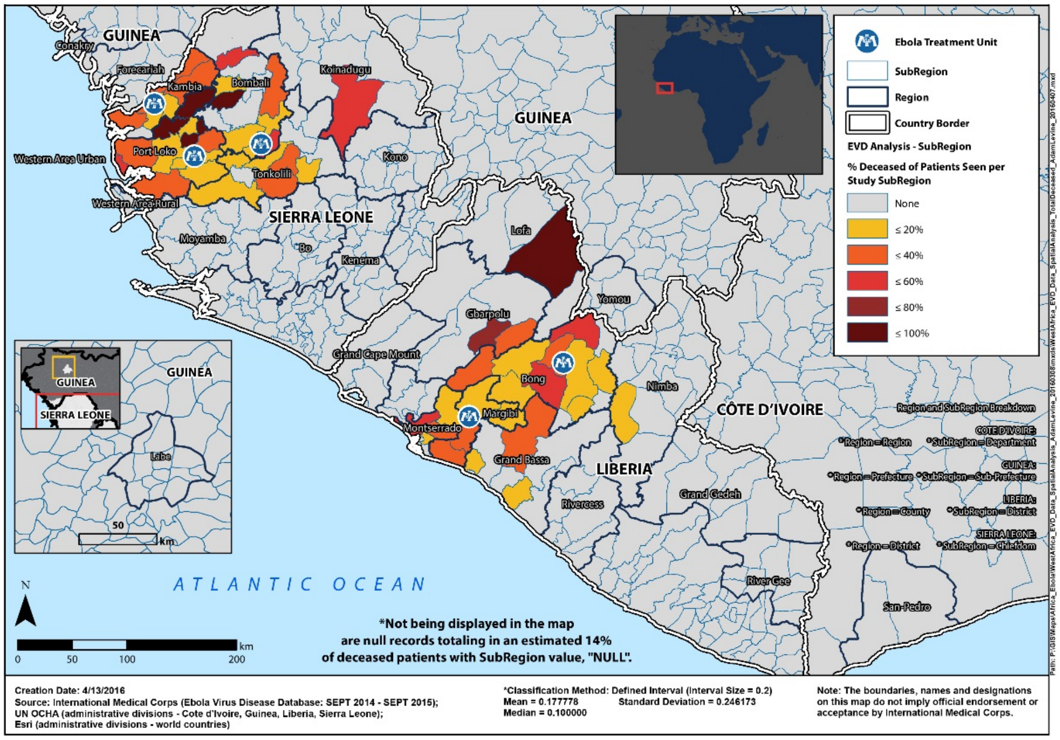

Roshania R, Mallow M, Dunbar N, Mansary D, Shetty P, Lyon T, et al. Successful implementation of a multicountry clinical surveillance and data collection system for Ebola virus disease in West Africa: findings and lessons learned. Glob Health Sci Pract. 2016;4(3). http://dx.doi.org/10.9745/GHSP-D-16-00186

SUPPLEMENTAL FIGURE 3.3. Proportion of EVD-Positive ETU Admissions Who Died by Patient Home Address, September 15, 2014, to September 15, 2015.

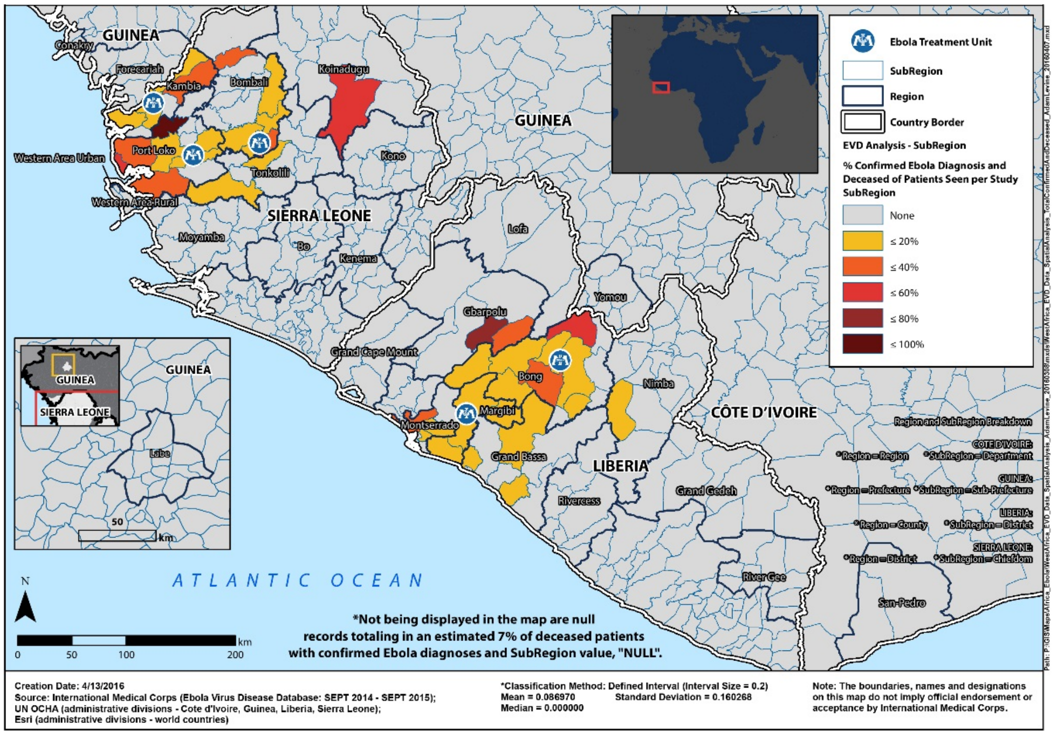

SUPPLEMENTAL FIGURE 3.4. Number of ETU Admissions by Location Where Patient Fell Ill, September 15, 2014, to September 15, 2015

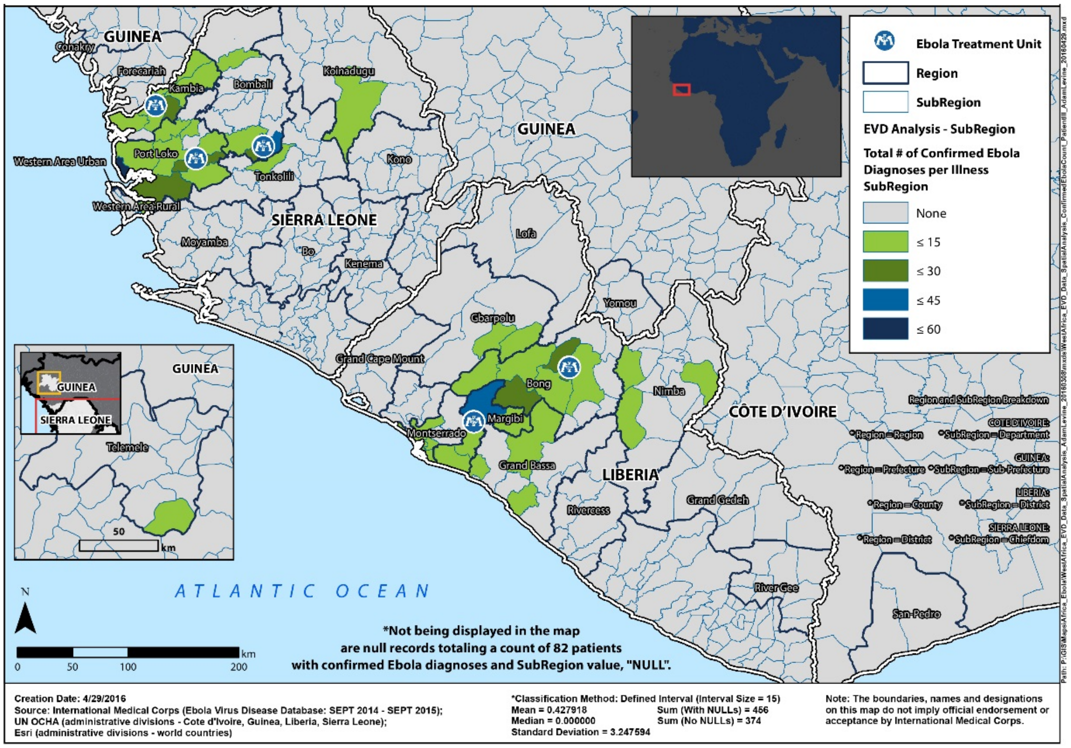

Roshania R, Mallow M, Dunbar N, Mansary D, Shetty P, Lyon T, et al. Successful implementation of a multicountry clinical surveillance and data collection system for Ebola virus disease in West Africa: findings and lessons learned. Glob Health Sci Pract. 2016;4(3). http://dx.doi.org/10.9745/GHSP-D-16-00186

SUPPLEMENTAL FIGURE 3.5. Number of ETU Admissions for Whom Illness Location Differed From Home Address, September 15, 2014, to September 15, 2015

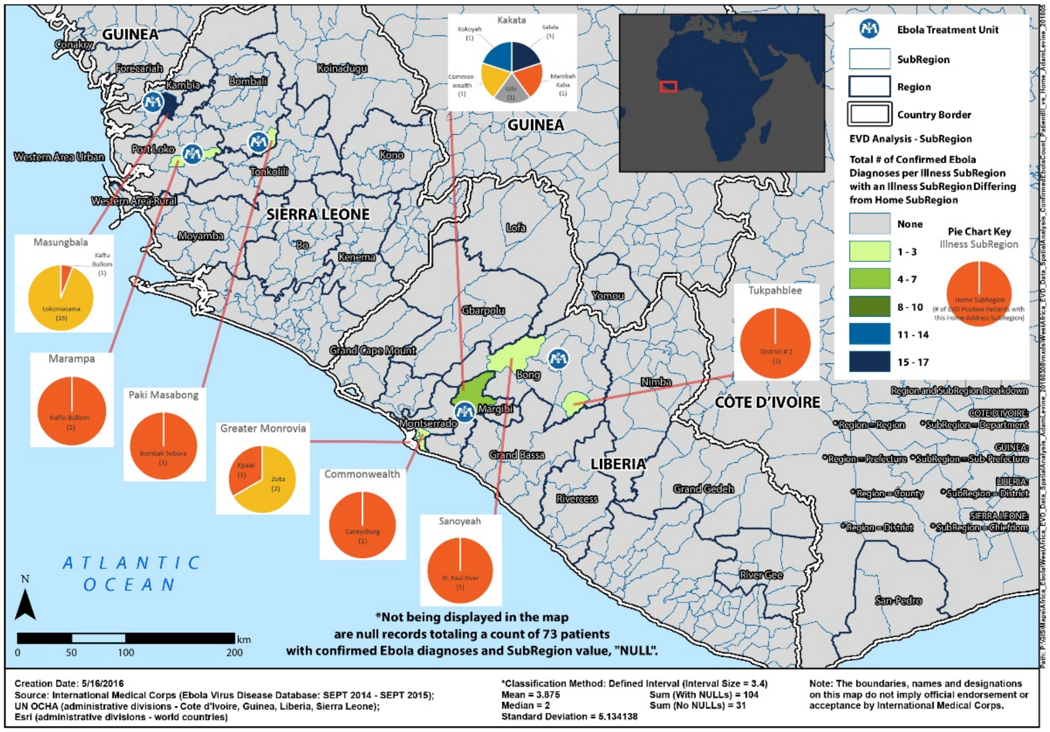

Supplement: supplementary material [file 16-00186-Levine-Supplementary-Material-3.pdf]
